# Combined Marbofloxacin-Geraniol-Tris-EDTA Exposure Delays Marbofloxacin Resistance Escalation in *Pseudomonas aeruginosa* While Early Genomic Changes Remain Limited

**DOI:** 10.3390/antibiotics15080754

**Published:** 2026-08-05

**Authors:** Mercédesz Adrienn Veres, Barbara Bodnár, Luca Takáts, Eszter Kaszab, Enikő Fehér, Ádám Kerek, Ákos Jerzsele

**Affiliations:** 1 Department of Pharmacology and Toxicology, University of Veterinary Medicine Budapest, István utca 2, H-1078 Budapest, Hungary; veres.a.mercedesz@univet.hu (M.A.V.); bodnar.barbara@student.univet.hu (B.B.); takats.luca@student.univet.hu (L.T.); jerzsele.akos@univet.hu (Á.J.); 2 National Laboratory of Infectious Animal Diseases, Antimicrobial Resistance, Veterinary Public Health and Food Chain Safety, University of Veterinary Medicine Budapest, István utca 2, H-1078 Budapest, Hungary; kaszab.eszter@univet.hu (E.K.); feher.eniko@univet.hu (E.F.); 3 Department of Bioinformatics, One Health Institute, Faculty of Health Sciences, University of Debrecen, Nagyerdei krt. 98, H-4032 Debrecen, Hungary; 4 Department of Microbiology and Infectious Diseases, University of Veterinary Medicine Budapest, István utca 2, H-1078 Budapest, Hungary; 5 National Laboratory of Virology, Szentágothai Research Centre, University of Pécs, H-7624 Pécs, Hungary

**Keywords:** *Pseudomonas aeruginosa*, marbofloxacin, geraniol, Tris-EDTA, biofilm, serial passaging, whole-genome sequencing, antimicrobial resistance

## Abstract

**Background**: *Pseudomonas aeruginosa* combines intrinsic resistance, adaptive plasticity, and biofilm formation, making treatment failure common in veterinary and human medicine. We examined whether geraniol and Tris-ethylenediaminetetraacetic acid (Tris-EDTA) modulate marbofloxacin activity during serial exposure and whether whole-genome sequencing detects genomic correlates of the observed phenotypic changes. **Methods**: A biofilm-producing reference *P. aeruginosa* ATCC 27853 strain was serially passaged for 10 days with marbofloxacin, geraniol, Tris-EDTA, and their double and triple combinations. Minimum inhibitory concentration (MIC) trajectories and fractional inhibitory concentration indices were determined. Efflux involvement was assessed by MIC testing with carbonyl cyanide 3-chlorophenylhydrazone (CCCP) in 22 canine isolates and the reference strain. Biofilm disruption was evaluated by crystal violet and MTS assays. Whole-genome sequencing was performed for day 0 controls and selected derivatives collected on days 2, 6, and 10. **Results**: Marbofloxacin alone showed a progressive MIC increase from 2 to 16 µg/mL, whereas its MIC remained low in the triple combination and in the marbofloxacin–Tris-EDTA combination. No antagonism was detected. The triple combination showed the most consistent interaction profile, meeting the prespecified synergy criterion on 7 of 10 passage days, compared with 5 of 10 days for marbofloxacin–Tris-EDTA, 2 of 10 days for geraniol–Tris-EDTA, and none for marbofloxacin–geraniol. CCCP caused at most a single twofold-dilution-step decrease in MIC, not supporting a dominant efflux-driven phenotype. In MTS assays, MTS-derived relative EC_50_ values approximated planktonic inhibitory concentrations, whereas crystal violet results were poorly reproducible. Draft genomes remained highly conserved, and only one candidate nonsynonymous sequence difference in *oprN* was observed in a day 2 marbofloxacin–Tris-EDTA derivative. **Conclusions**: Geraniol- and especially Tris-EDTA-containing combinations constrained in vitro marbofloxacin resistance escalation. The marked phenotypic separation despite minimal genomic divergence is consistent with a possible contribution from regulatory or transcriptional adaptation, although this mechanism requires direct experimental confirmation.

## 1. Introduction

*Pseudomonas aeruginosa* is a high-priority opportunistic pathogen whose clinical relevance stems from the convergence of low outer-membrane permeability, inducible and constitutive efflux activity, enzymatic resistance, and efficient adaptive responses under antimicrobial pressure [1,2,3]. In addition to its multidrug-resistant phenotype, its ability to establish biofilms creates a second layer of tolerance that further compromises therapeutic success in chronic and device-associated infections [4,5,6].

In companion-animal medicine, *P. aeruginosa* is a particularly problematic secondary pathogen in canine otitis externa, where chronicity, tissue irritation, repeated topical exposure, and biofilm formation can select for difficult-to-eradicate populations [7,8,9]. The same organism is also of major importance in human medicine, including nosocomial infections and cystic fibrosis-associated airway disease, underscoring the translational relevance of veterinary models and One Health-oriented resistance research [10,11,12,13].

The importance of integrating phenotypic susceptibility testing with genomic or marker-based characterization is also evident from recent veterinary studies of poultry-associated Escherichia coli and Proteus mirabilis and of Pasteurella multocida from multiple host species [14,15,16].

Fluoroquinolones remain important antipseudomonal agents in veterinary practice, and marbofloxacin is often considered one of the more active members of this class against companion-animal Pseudomonas isolates [17,18,19]. However, fluoroquinolone efficacy may erode rapidly through target-site mutations, altered membrane permeability, efflux upregulation, and adaptive physiological switching under repeated exposure [3,20,21,22]. This raises a key stewardship question: can rational non-antibiotic adjuvants slow phenotypic resistance escalation during repeated exposure even if they do not function as classical bactericidal partners on their own? This question also fits within broader veterinary stewardship efforts, including recent Hungarian work emphasizing structured antimicrobial use and systematic monitoring as complementary routes to reducing antimicrobial exposure [23,24].

Two such adjuvant candidates are geraniol and Tris-ethylenediaminetetraacetic acid (Tris-EDTA). Geraniol, a plant-derived monoterpene alcohol, has been linked to membrane perturbation, quorum-sensing attenuation, and suppression of *P. aeruginosa* virulence-associated traits [25,26,27,28,29]. Tris-EDTA combines buffering and cation-chelating effects that destabilize the Gram-negative envelope, increase permeability, and may potentiate antibiotic activity against resistant pseudomonads [10,30,31]. Both mechanisms are highly relevant in the context of biofilm-rich infections and may be particularly attractive for topical veterinary formulations. Complementary veterinary approaches to AMR control increasingly include non-antibiotic antimicrobial systems and interventions targeting population-level bacterial communication. Stabilized hypochlorous acid has been discussed as a non-antibiotic antimicrobial tool, while quorum-sensing modulation has been highlighted as a potential means of influencing collective stress responses and resistance trajectories [32,33].

Yet an important mechanistic gap remains. Phenotypic stabilization of susceptibility under combination exposure does not necessarily imply rapid accumulation of readily detectable genomic changes. Early adaptation may instead arise from transient or semi-stable regulatory shifts, altered expression of intrinsic resistance systems, or biofilm-associated physiological restructuring that is not fully captured by static genome comparison [34,35,36]. A framework that combines serial phenotyping, antibiofilm testing, efflux interrogation, and genome-level screening is therefore useful for distinguishing what the phenotype clearly shows from what the genome can already explain.

In the present study, we integrated serial MIC passaging, fractional inhibitory concentration (FIC)-based interaction analysis, CCCP-assisted efflux testing, antibiofilm assays, and whole-genome sequencing to evaluate the marbofloxacin-geraniol-Tris-EDTA system in *P. aeruginosa*. We hypothesized that combinations containing geraniol and/or Tris-EDTA would attenuate marbofloxacin resistance escalation during repeated exposure and that genomic screening would reveal at least part of the mechanistic basis of this phenotype. The central question was not only whether synergy could be demonstrated, but also whether the observed phenotypic protection would leave a clear and treatment-linked genomic footprint.

## 2. Results

### 2.1. Serial Passaging Identified Marked Divergence Between Marbofloxacin Alone and Combination Regimens

**Figure 1:**
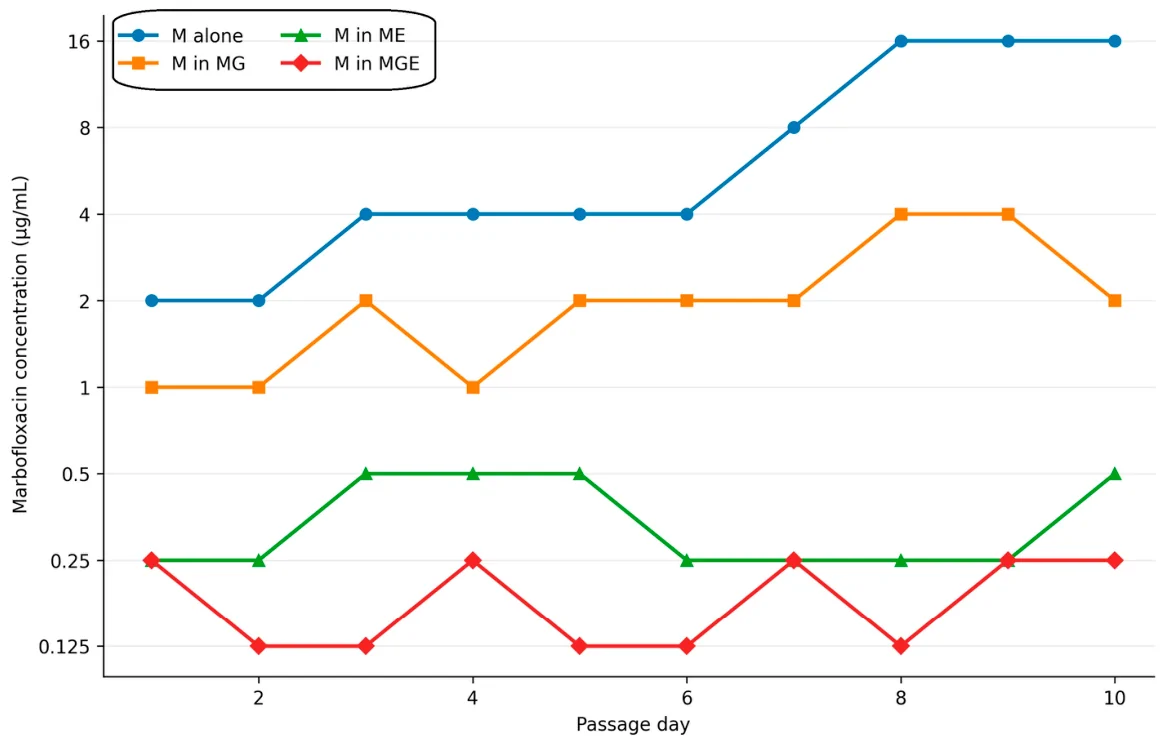
Trajectory of the marbofloxacin component across serial passages. Marbofloxacin monotherapy showed progressive upward drift, whereas the marbofloxacin component remained low under marbofloxacin-geraniol (MG), marbofloxacin-Tris-EDTA (ME), and especially marbofloxacin-geraniol-Tris-EDTA (MGE) exposure. The *y*-axis is shown on a base-2 logarithmic scale to reflect dilution-based minimum inhibitory concentration (MIC) progression.

During 10 days of serial passage, marbofloxacin alone showed a stepwise MIC increase from 2 to 16 µg/mL, representing an eightfold escalation. By contrast, the marbofloxacin component remained substantially lower when the drug was combined with Tris-EDTA (0.25–0.5 µg/mL for most passages), with geraniol (1–4 µg/mL), or with both adjuvants in the triple combination (0.125–0.25 µg/mL throughout). Geraniol and Tris-EDTA alone displayed only minor fluctuations over time. The steepest MIC escalation was observed under marbofloxacin monotherapy, whereas combination regimens, particularly those containing Tris-EDTA, showed substantially lower marbofloxacin MIC trajectories (Figure 1; Table 1).

The triple combination was particularly notable because the marbofloxacin component never approached the monotherapy trajectory despite repeated passaging. On day 10, the marbofloxacin component concentration required during MGE exposure (0.25 µg/mL) was 64-fold lower than the concentration required during marbofloxacin monotherapy (16 µg/mL). This comparison refers to the marbofloxacin component concentrations within the respective exposure regimens and should not be interpreted as an equivalent difference in the intrinsic marbofloxacin susceptibility of the recovered derivatives. The complete 10-day MIC trajectories for all single-agent and combination regimens are shown in Table 1. The complete day-by-day MIC trajectories and corresponding FIC values are provided in Supplementary Table S1.

### 2.2. Interaction Analysis Showed No Antagonism and Recurrent Synergy in Tris-EDTA-Containing Combinations

FICI analysis identified no antagonistic interactions during the passaging experiment. The marbofloxacin–Tris-EDTA combination (ME) met the prespecified synergy criterion (FICI ≤ 0.5) on 5 of 10 passage days, whereas geraniol–Tris-EDTA (GE) met this criterion on 2 of 10 days. Marbofloxacin–geraniol (MG) remained within the no-interaction range throughout the experiment.

**Figure 2:**
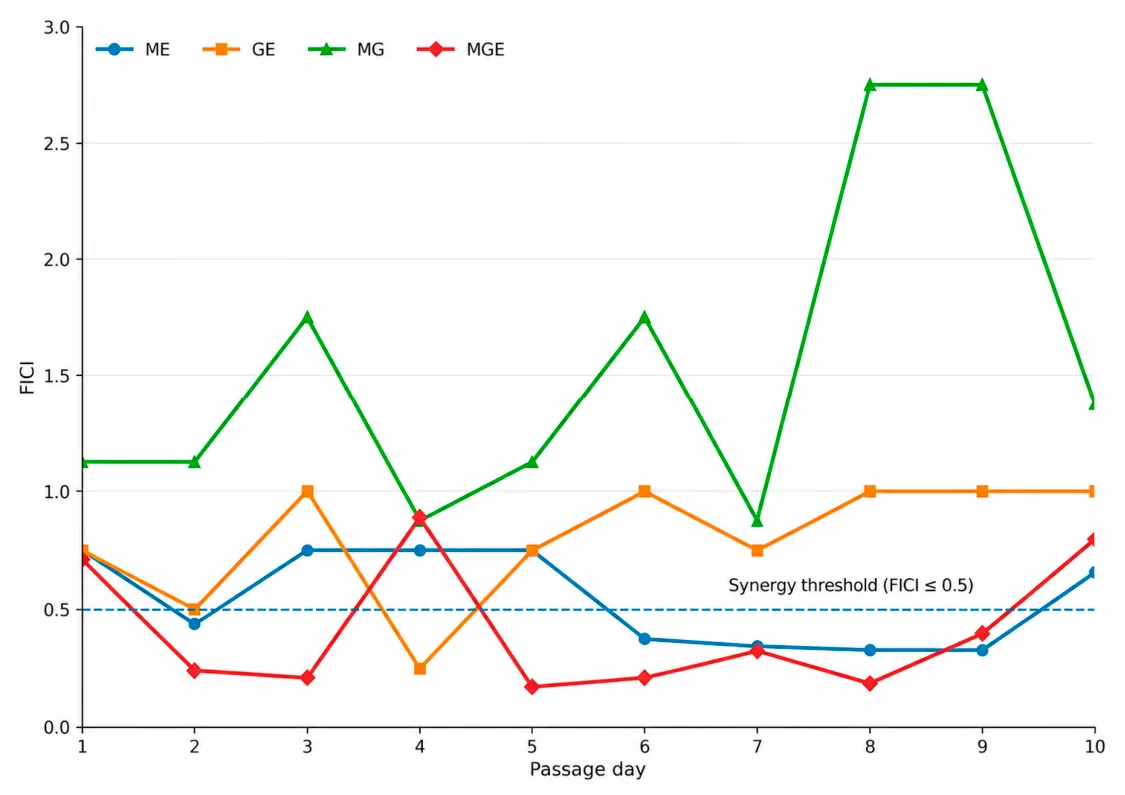
Fractional inhibitory concentration index (FICI) trajectories of the evaluated combinations during serial passaging. The dashed line indicates the prespecified threshold for synergy (FICI ≤ 0.5). Values > 0.5 to ≤4 were interpreted as no interaction, whereas values >4 were considered antagonistic. No antagonistic interaction was observed during the experiment. ME, marbofloxacin + Tris-EDTA; GE, geraniol + Tris-EDTA; MG, marbofloxacin + geraniol; MGE, marbofloxacin + geraniol + Tris-EDTA.

The triple combination (MGE) showed the strongest overall interaction profile, meeting the synergy criterion on 7 of 10 passage days. The remaining three MGE observations fell within the no-interaction range, and no antagonistic interaction was detected for any combination (Figure 2; Table 2).

### 2.3. CCCP Testing Did Not Support Dominant Efflux-Mediated Resistance Under the Applied Interpretive Criteria

CCCP-assisted MIC testing was performed on 22 canine clinical isolates and the *Pseudomonas aeruginosa* (ATCC 27853) reference strain to assess whether the observed resistance phenotype was strongly efflux-dependent. Across the single-agent assays, marbofloxacin showed only occasional one-dilution MIC reductions in the presence of CCCP, geraniol showed no change, and Tris-EDTA showed a uniform but still limited one-step reduction in most isolates. Similar patterns were observed for the double and triple combinations. One-step MIC reductions were observed for marbofloxacin in 5 of 23 strains, Tris-EDTA in 21 of 23, GE in 7 of 23, MG in 3 of 23, and ME in 2 of 23. No MIC reduction was observed for geraniol or MGE. Isolate-level values are provided in Supplementary Table S2.

**Figure 3:**
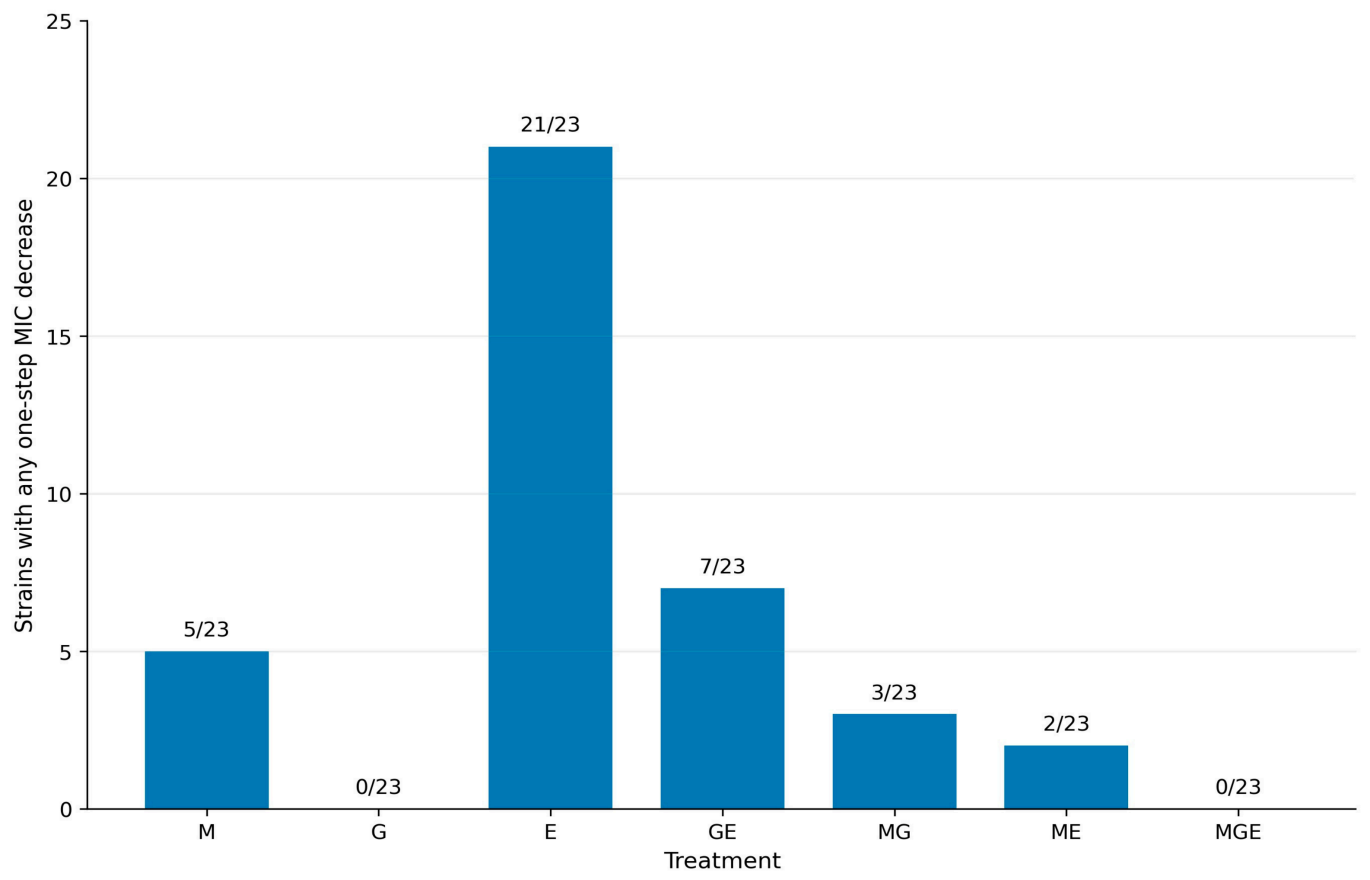
Number of strains showing any one-step decrease in minimum inhibitory concentration (MIC) in the presence of carbonyl cyanide 3-chlorophenylhydrazone (CCCP). Counts are shown for 22 canine clinical isolates and the *Pseudomonas aeruginosa* ATCC 27853 reference strain. No isolate–treatment pair exhibited a ≥4-fold MIC reduction. M, marbofloxacin; G, geraniol; E, Tris-EDTA; ME, marbofloxacin + Tris-EDTA; GE, geraniol + Tris-EDTA; MG, marbofloxacin + geraniol; MGE, marbofloxacin + geraniol + Tris-EDTA.

No isolate–treatment pair showed a ≥4-fold MIC reduction in the presence of CCCP. Accordingly, the assay did not identify a prevalent high-magnitude CCCP-responsive phenotype across the tested clinical isolate panel under the applied conditions (Figure 3; Table 3). Because the passage-derived lineages were not individually examined with CCCP, these results do not directly exclude a contribution of efflux regulation to the serial-passaging trajectories.

### 2.4. MTS-Based Antibiofilm Testing Tracked Inhibitory Concentrations, Whereas Crystal Violet Measurements Were Poorly Reproducible

The crystal violet assay showed substantial between-replicate variability and did not support robust concentration–response estimation. By contrast, the MTS assay yielded interpretable curves, and the MTS-derived relative EC_50_ (rEC_50_) values generally approximated the corresponding planktonic MICs. A direct comparison is provided in Supplementary Table S3.

In the present reference-strain model, the concentrations required to reduce biofilm-associated metabolic activity were broadly similar to those required to inhibit planktonic growth. The data did not demonstrate preferential activity of the combinations against established biofilms.

### 2.5. Draft Genome Assemblies Remained Highly Conserved Across Selected Passage-Derived Genomes

**Figure 4:**
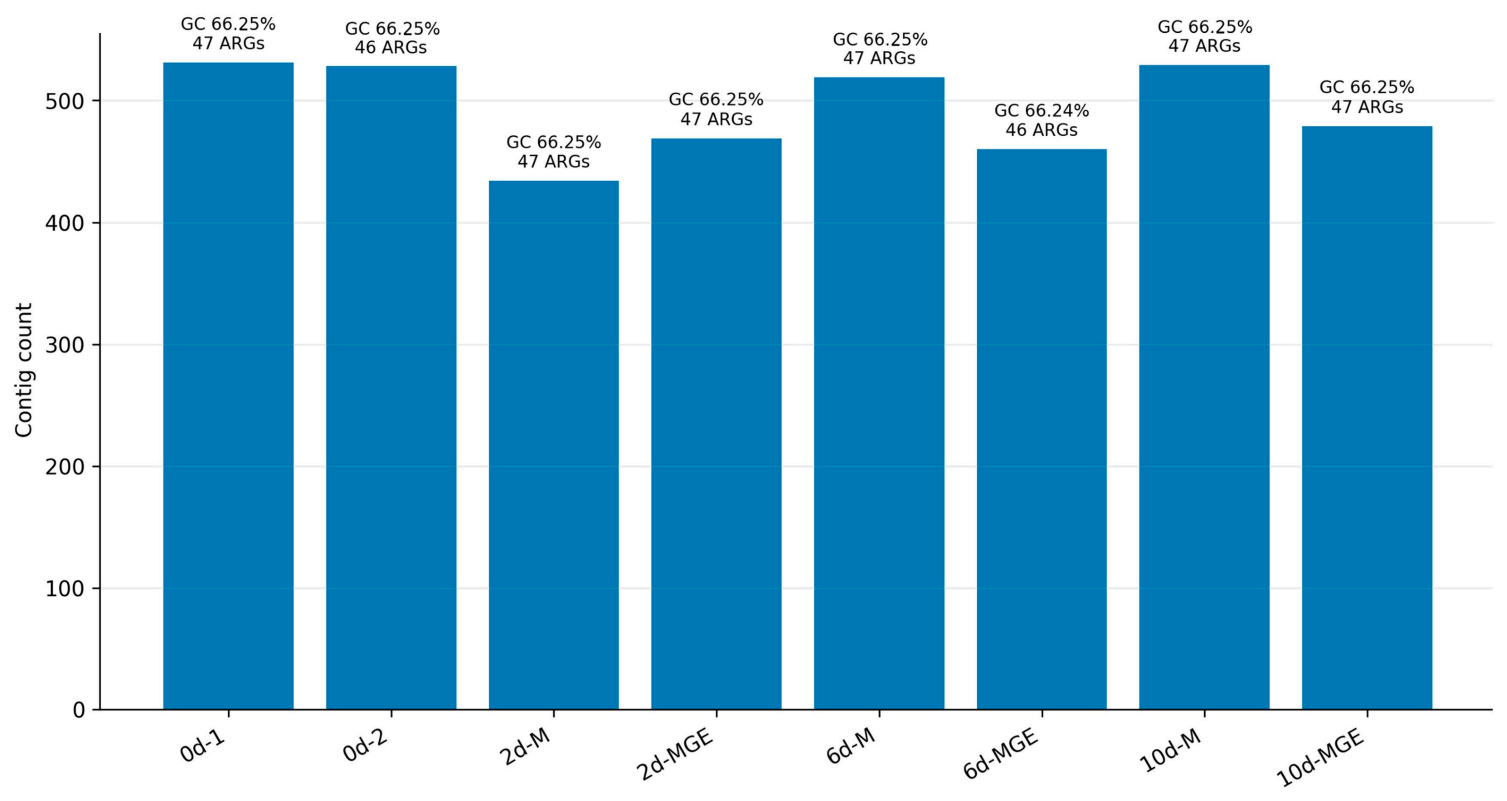
Assembly-level characteristics of eight representative sequenced samples. Contig counts varied within a relatively narrow range, whereas GC content and the number of detected resistance genes remained nearly invariant across the selected day 0, day 2, day 6, and day 10 genomes. M, marbofloxacin; MGE, marbofloxacin + geraniol + Tris-EDTA.

Whole-genome sequencing was performed on 23 samples. Assembly metrics for all sequenced samples are provided in Supplementary Table S4, whereas eight representative day 0, day 2, day 6, and day 10 genomes are summarized in Table 4 and Figure 4. Across the selected genomes, total assembly lengths clustered around 6.78 Mb and GC content remained essentially invariant at approximately 66.24–66.25%. The number of detected resistance genes also varied minimally, with 46–47 detected genes per sample. Assembly metrics and resistome-level summary features of the sequenced derivatives are presented in Supplementary Table S4.

No broad genome-scale differences were evident at the level of assembly metrics or, overall, the number of detected resistance genes among the selected sequenced derivatives (Figure 4; Table 4).

### 2.6. Genome-Level Screening Identified Only Minimal Treatment-Linked Variation in Fluoroquinolone-Associated Determinants

Across the 23 sequenced derivatives, 19 fluoroquinolone-associated resistance genes were screened in detail. Most showed stable coverage and identity relative to CARD-derived references. Genes showing complete identity and coverage relative to the selected reference sequence were not subjected to further manual inspection for coding differences. Following exclusion of the incompletely covered mexY locus, read-based comparison with the day 0 parental genome identified one candidate nonsynonymous variant in *oprN* in the day 2 ME derivative (c.311A>G; p.Asn104Ser; read depth, 54×; alternate-allele frequency, 98.5%). The variant was not detected in either day 0 control or in the other sequenced derivatives (Table 5). A consolidated summary of the principal genome-level observations is provided in Supplementary Table S5.

Treatment-linked coding variation within the screened fluoroquinolone-associated loci was limited. These findings did not support rapid accumulation of structural mutations in the screened fluoroquinolone-associated loci as the primary explanation for the phenotypic separation observed during passaging.

## 3. Discussion

A clear phenotypic separation between marbofloxacin monotherapy and adjuvant-containing regimens emerged during serial passaging. Marbofloxacin alone showed progressive MIC escalation, while Tris-EDTA-containing regimens, particularly MGE, markedly restrained this drift. The strongest signal in the dataset was therefore phenotypic rather than genomic.

Interaction profiles also differed among regimens. MG did not meet the prespecified synergy criterion on any passage day; GE, ME, and MGE met it on 2, 5, and 7 passage days, respectively. The recurrent synergy observed with ME and MGE indicates that Tris-EDTA contributed more consistently than geraniol to preserving marbofloxacin activity in this experimental setting [10,30]. The favorable MGE profile suggests that geraniol may still provide additional biological support when layered onto the permeability-modulating effect of Tris-EDTA, even though its independent interaction with marbofloxacin was less consistent.

This hierarchy is biologically plausible. Tris-EDTA can destabilize the Gram-negative outer envelope through divalent-cation chelation and thereby improve antimicrobial access to periplasmic and intracellular targets [10,30,31]. Geraniol, by contrast, may exert a broader but less directly bactericidal modulatory role through membrane interaction, quorum-sensing attenuation, and suppression of virulence-linked physiology [25,26,29]. Within a serial-passaging framework, these distinct modes of action could translate into different capacities to prevent selection for higher marbofloxacin tolerance.

CCCP testing further defined the mechanistic limits of the present data. *P. aeruginosa* is intrinsically rich in multidrug efflux systems, and efflux upregulation is a classic route toward reduced fluoroquinolone susceptibility [20,22]. Nevertheless, the assay did not show the ≥4-fold MIC reductions expected for strong efflux-mediated interpretation. This does not exclude a contribution of efflux; rather, it suggests that efflux alone is unlikely to be the dominant explanatory layer for the observed resistance-delay phenotype under these assay conditions. Subtler regulatory effects, transient shifts in pump expression, or pathway interactions below the chosen interpretive threshold may still be involved. Moreover, the CCCP screen was performed primarily in an independent clinical isolate panel rather than in each passage-derived lineage; therefore, it provides contextual evidence on CCCP responsiveness but cannot directly assign the mechanism underlying the serial-passaging trajectories.

Whole-genome sequencing sharpened this interpretation further. Genome size, GC content, contig architecture, and total resistance-gene burden were highly stable across the selected derivatives. The screened fluoroquinolone-associated determinants showed almost complete conservation, and only one nonsynonymous *oprN* change was identified in a single day 2 ME derivative. That isolated event may be biologically interesting, but it is clearly insufficient to explain the global pattern of resistance stabilization across ME and MGE lineages. Taken together, these findings suggest that phenotypic divergence emerged faster than stable structural genomic divergence in the loci surveyed here.

Importantly, the observed phenotype–genotype asymmetry is itself biologically informative. Short-term adaptive trajectories in *P. aeruginosa* may involve regulatory plasticity, altered gene expression, membrane-state remodelling, quorum-sensing responses, and biofilm-associated physiological changes that are not captured by static draft-genome comparison [34,36]. These findings motivate transcriptomic follow-up at matched passage points but do not, by themselves, establish a regulatory mechanism.

Biofilm assays added a further nuance to the overall findings. In the MTS assay, EC_50_ values largely tracked planktonic MIC values instead of revealing a strikingly lower biofilm-specific effective window for combinations. The combinations therefore did not show a markedly lower effective window against established biofilms. Their principal benefit in this model may instead lie in restricting adaptation and preserving antibacterial activity during repeated exposure. The poor reproducibility of crystal violet in this dataset is also consistent with the known limitations of biomass-only assays in some Pseudomonas systems [37].

From a translational perspective, these findings are relevant to topical antipseudomonal strategy development for canine otitis externa, where repeated local exposure, chronic inflammation, and biofilm-rich microenvironments create ideal conditions for progressive loss of susceptibility during therapy [8,38]. A combination that limits resistance escalation while maintaining low marbofloxacin-component concentrations may warrant further evaluation as a topical strategy. However, translational relevance depends not only on antimicrobial activity but also on local host-tissue tolerance. At the same time, this potential should be discussed cautiously because the study used a reference strain for the sequencing arm and an in vitro exposure design rather than an infection model.

Available data indicate that host-cell effects of these adjuvants are strongly concentration- and model-dependent. Geraniol showed no detectable cytotoxicity at 0.125% in Vero E6 or L929 cells after 1 h of exposure, whereas 0.25% geraniol produced measurable cytotoxicity in both cell lines, with a markedly greater effect in Vero E6 cells [39]. Direct mammalian-cell cytotoxicity thresholds specifically for Tris-buffered EDTA are less well characterized. In a fibroblast model, a 17% EDTA irrigant diluted to 0.5% (*v*/*v*) in culture medium reduced short-term cell viability, whereas the same preparation diluted to 0.1% did not differ from the control under the conditions tested [40]. Conversely, repeated administration of a canine otic formulation containing 1.21 mg/mL disodium EDTA in Tris buffer together with 0.22 mg/mL polyhexamethylene biguanide produced no detectable vestibular or auditory toxicity, including after myringotomy [41]. These observations cannot be directly extrapolated to the geraniol–Tris-EDTA regimen evaluated here and do not establish the safety of the combined formulation. In particular, additive or synergistic host-cell toxicity resulting from simultaneous geraniol and Tris-EDTA exposure was not assessed in the present study. Dedicated cytotoxicity studies in relevant canine epithelial or keratinocyte models, followed by local tolerance studies, will therefore be required before therapeutic formulation or in vivo application.

Several limitations define the scope of these findings. First, the sequencing work focused on one lineage background and selected passage points rather than dense temporal sampling. Second, the genomic analyses were based on draft assemblies and targeted interpretation of known resistance-associated loci; therefore, subtle structural variants, regulatory mutations outside the screened set, or epigenetic effects may have been missed. Third, no transcriptomic or proteomic layer was available to test whether the conserved genomes nonetheless diverged strongly at the level of gene expression. Fourth, the antibiofilm evaluation was stronger metabolically than structurally because crystal violet reproducibility was suboptimal. In addition, the serial-passaging experiment included one independently passaged lineage per regimen. Passage days within a lineage were therefore not independent biological replicates, and the trajectories should be considered exploratory until confirmed in multiple independently evolved lineages. The CCCP experiment was performed primarily in clinical isolates rather than in the individual passage-derived lineages and therefore cannot directly explain the serial-passaging phenotype. In addition, short-read draft assemblies limit the resolution of plasmid structures, mobile-element linkage, repeat-associated variants, and other complex structural changes.

Overall, marbofloxacin monotherapy produced a pronounced upward MIC trajectory, whereas Tris-EDTA-containing combinations maintained lower marbofloxacin-component MICs. Neither the CCCP screen nor the short-read genomic comparison provided a direct mechanistic explanation for this difference. Confirmation in independently passaged lineages and direct analysis of gene expression will therefore be required.

## 4. Materials and Methods

### 4.1. Study Design

The study integrated four connected experimental modules: serial MIC passaging of a biofilm-producing *P. aeruginosa* ATCC 27853 reference strain under single-agent, double-combination, and triple-combination exposure; interaction analysis by FIC indexing; CCCP-assisted MIC testing of 22 canine clinical isolates plus the reference strain; and whole-genome sequencing of selected passage-derived derivatives. The overall analytical aim was to relate phenotypic resistance trajectories to genome-level signatures.

### 4.2. Antimicrobial Agents and Combinations

Marbofloxacin (M), geraniol (G), and Tris-EDTA were evaluated alone and in the following combinations: marbofloxacin + Tris-EDTA (ME), geraniol + Tris-EDTA (GE), marbofloxacin + geraniol (MG), and marbofloxacin + geraniol + Tris-EDTA (MGE). Starting concentrations in the first well of the serial twofold dilution series were 16 µg/mL for marbofloxacin, 6.4 mg/mL for geraniol, and 25.6 mg/mL for Tris-EDTA. For combination regimens, first-well concentrations were 8 µg/mL + 16 mg/mL for ME, 3.2 mg/mL + 3.2 mg/mL for GE, 4 µg/mL + 4 mg/mL for MG, and 2 µg/mL + 2.3 mg/mL + 4.6 mg/mL for MGE.

### 4.3. Serial Passaging and MIC Determination

A biofilm-producing *P. aeruginosa* ATCC 27853 reference strain was serially passaged for 10 consecutive days in 96-well microtiter plates. MIC testing was performed by twofold broth microdilution in cation-adjusted Mueller–Hinton broth (CAMHB; Sigma-Aldrich, St. Louis, MO, USA) following CLSI M07 principles, with adaptations for the serial-passaging design. The final bacterial inoculum was approximately 5 × 10^5^ CFU/mL, and plates were incubated aerobically at 35 ± 2 °C for 18–24 h. Growth and sterility controls were included on each plate. The MIC was defined as the lowest concentration showing no visible bacterial growth. For daily passaging, an aliquot was transferred from the first well showing visible growth immediately below the MIC endpoint into a freshly prepared dilution series for the subsequent passage.

### 4.4. Combination Interaction Analysis

For each passage day, the fractional inhibitory concentration index (FICI) was calculated as the sum of the MIC of each component in combination divided by the MIC of the same component tested alone. For two-component combinations, FICI was calculated as FICI = FICA + FICB, whereas for the triple combination it was calculated as FICI = FICA + FICB + FICC. Values ≤ 0.5 were interpreted as synergy, values > 0.5 to ≤4 as no interaction, and values > 4 as antagonism. Because interpretation of FICI values for three-component combinations is less standardized than for two-component combinations, the MGE trajectory was treated as a descriptive interaction profile [42].

### 4.5. CCCP-Assisted Efflux Interrogation

Potential energy-dependent efflux activity was examined using carbonyl cyanide 3-chlorophenylhydrazone (CCCP) in 22 canine clinical isolates and the *P. aeruginosa* ATCC 27853 reference strain. MICs were determined under otherwise identical conditions in the presence and absence of CCCP. CCCP was applied at a final concentration of 104 µg/mL. This concentration was selected on the basis of preliminary growth-control testing because CCCP at 104 µg/mL did not inhibit bacterial growth when applied alone, thereby minimizing confounding by a direct antimicrobial effect of the inhibitor. Matched solvent and CCCP-only growth controls were included. A ≥4-fold reduction in MIC in the presence of CCCP was considered evidence consistent with a substantial contribution of energy-dependent efflux. Because CCCP disrupts the proton motive force rather than inhibiting a specific efflux system, the assay was interpreted as a phenotypic screening method rather than as pump-specific mechanistic confirmation.

### 4.6. Biofilm Disruption Assays

Biofilm response was evaluated using 24 h biofilms formed by *P. aeruginosa* ATCC 27853 in 96-well microtiter plates. After removal of the planktonic supernatant, established biofilms were exposed for an additional 24 h to twofold serial dilutions of marbofloxacin, geraniol, Tris-EDTA, or their double and triple combinations over concentration ranges corresponding to those used in the planktonic susceptibility assays. Each concentration was tested in three parallel wells.

Attached biofilm biomass was assessed using a modified crystal violet assay. Following treatment, wells were washed to remove non-adherent cells, stained with 0.1% crystal violet, washed again, and the retained dye was solubilized before absorbance measurement at 595 nm [43]. Biofilm-associated metabolic activity was evaluated using the CellTiter 96 AQueous One Solution Cell Proliferation Assay (Promega, Madison, WI, USA). The MTS reagent was added according to the experimental protocol, plates were incubated for 1.5 h at 37 °C, and absorbance was measured at 492 nm [44].

Concentration–response relationships were analyzed by four-parameter logistic nonlinear regression. The reported relative EC_50_ (rEC_50_) was defined as the concentration corresponding to the midpoint between the fitted upper and lower response plateaus. It therefore does not represent an absolute 50% reduction relative to untreated control. Relative EC_50_ estimates were interpreted only when the concentration range adequately defined the upper and lower portions of the fitted response curve. Because crystal violet measurements showed substantial between-replicate variability, MTS-derived rEC_50_ values were used as the principal quantitative biofilm-response endpoint [45,46].

### 4.7. DNA Extraction, Sequencing, and Bioinformatics

A total of 23 samples, comprising day 0 controls and selected early-, intermediate-, and late-passage derivatives, were subjected to whole-genome sequencing. Genomic DNA was extracted using the Quick-DNA Fungal/Bacterial Miniprep Kit (Zymo Research, Irvine, CA, USA). Libraries were prepared using the Nextera XT DNA Library Preparation Kit and Nextera XT Index Kit v2 (Illumina, San Diego, CA, USA), following the reduced-volume workflow described by Bali et al. [47], with project-specific indexing. Pooled libraries were sequenced on an Illumina NovaSeq 6000 using a NovaSeq 6000 v1.5 Reagent Kit to generate paired-end 2 × 151 bp reads.

Raw-read quality was assessed using FastQC v0.11.9. Adapter removal and quality filtering were performed using fastp v0.23.2, followed by read-error correction with Bloocoo v1.0.7 [48,49,50]. Reads were assembled independently using SPAdes v4.3.0 and MEGAHIT v1.2.9, and the resulting assemblies were merged using GAM-NGS v1.1b [51,52,53]. Assembly quality was assessed using QUAST v5.2 [54], and taxonomic identity was confirmed using Kraken2 v2.17.1 [55] with the PrackenDB database (built on January 2026). Antimicrobial-resistance genes were screened using ABRicate v1.0.1 against CARD version 4.0.0 [20,56], applying minimum identity and coverage thresholds of 80% and 80%, respectively. Virulence-associated genes were screened against VFDB version 2026 using the same predefined thresholds [57]. Prophage regions were assessed using PHASTEST [58], plasmid-associated contigs were predicted using PlasFlow v1.1 [59], and mobile genetic elements were screened using MobileElementFinder v1.0.3 with database version 2020-06-09 [60].

Read-based comparison with the day 0 parental genome identified one candidate treatment-associated nonsynonymous variant in *oprN* in the day 2 ME derivative (c.311A>G; p.Asn104Ser; read depth, 54×; alternate-allele frequency, 98.5%). The variant was not detected in either day 0 control or in the other sequenced derivatives.

### 4.8. Statistical Approach

Data handling and descriptive analyses were performed in R version 4.3.1. Serial-passaging trajectories were summarized descriptively because sequential passage days represented repeated observations within the same experimental lineage and were not statistically independent. Technical replicate wells were not treated as independent biological replicates. FICI profiles, CCCP-associated MIC shifts, and genomic screening outputs were summarized using counts, medians, ranges, and fold differences, as appropriate. MTS concentration–response curves were fitted using four-parameter logistic nonlinear regression, and rEC_50_ values were derived from the fitted upper and lower response plateaus. No confirmatory inferential comparison among serial-passaging trajectories was performed.

## 5. Conclusions

Serial exposure to marbofloxacin-containing combinations produced markedly different MIC trajectories from marbofloxacin monotherapy in *P. aeruginosa* ATCC 27853. Tris-EDTA-containing regimens, particularly the triple combination, maintained lower marbofloxacin-component MICs throughout the experiment and showed the most favorable FICI profiles. In parallel, the sequenced passage-derived genomes remained highly conserved, and only limited candidate coding variation was detected in the screened fluoroquinolone-associated loci. This phenotype–genotype divergence is consistent with a possible contribution from regulatory or transcriptional adaptation, but this mechanism remains to be tested directly. The findings support further validation across independently evolved lineages, clinical isolates, and transcriptome-resolved experimental designs.

## Figures and Tables

**Table 1:** Day-by-day minimum inhibitory concentration (MIC) trajectories during 10-day serial passaging of *Pseudomonas aeruginosa* ATCC 27853 under single-agent and combination exposure.

Regimen	Day 1	Day 2	Day 3	Day 4	Day 5	Day 6	Day 7	Day 8	Day 9	Day 10
M (µg/mL)	2	2	4	4	4	4	8	16	16	16
G (mg/mL)	1.6	1.6	1.6	1.6	3.2	1.6	3.2	1.6	1.6	1.6
E (mg/mL)	0.8	1.6	1.6	1.6	1.6	1.6	1.6	1.6	1.6	1.6
ME (M + E)	0.25 + 0.5	0.25 + 0.5	0.5 + 1	0.5 + 1	0.5 + 1	0.25 + 0.5	0.25 + 0.5	0.25 + 0.5	0.25 + 0.5	0.5 + 1
GE (G + E)	0.4 + 0.4	0.4 + 0.4	0.8 + 0.8	0.2 + 0.2	0.8 + 0.8	0.8 + 0.8	0.8 + 0.8	0.8 + 0.8	0.8 + 0.8	0.8 + 0.8
MG (M + G)	1 + 1	1 + 1	2 + 2	1 + 1	2 + 2	2 + 2	2 + 2	4 + 4	4 + 4	2 + 2
MGE (M + G + E)	0.25 + 0.287 + 0.325	0.125 + 0.123 + 0.162	0.125 + 0.123 + 0.162	0.25 + 0.287 + 0.325	0.125 + 0.123 + 0.162	0.125 + 0.123 + 0.162	0.25 + 0.287 + 0.325	0.125 + 0.123 + 0.162	0.25 + 0.287 + 0.325	0.25 + 0.287 + 0.325

Abbreviations: M, marbofloxacin; G, geraniol; E, Tris-EDTA; ME, marbofloxacin + Tris-EDTA; GE, geraniol + Tris-EDTA; MG, marbofloxacin + geraniol; MGE, marbofloxacin + geraniol + Tris-EDTA.

**Table 2:** Fractional inhibitory concentration index profiles of the evaluated combinations across the 10-day serial-passaging experiment. ME, marbofloxacin + Tris-EDTA; GE, geraniol + Tris-EDTA; MG, marbofloxacin + geraniol; MGE, marbofloxacin + geraniol + Tris-EDTA.

Combination	Median FICI	Days with Synergy, FICI ≤ 0.5	Days Without Synergy, 0.5 < FICI ≤ 4	Antagonistic DAYS, FICI > 4
ME	0.55	5/10	5/10	0/10
GE	0.78	2/10	8/10	0/10
MG	1.25	0/10	10/10	0/10
MGE	0.36	7/10	3/10	0/10

**Table 3:** Summary of CCCP-associated minimum inhibitory concentration (MIC) shifts. M, marbofloxacin; G, geraniol; E, Tris-EDTA; ME, marbofloxacin + Tris-EDTA; GE, geraniol + Tris-EDTA; MG, marbofloxacin + geraniol; MGE, marbofloxacin + geraniol + Tris-EDTA.

Treatment	Pairs with any MIC Decrease	Maximum Observed Shift	Interpretation
M	5/23	1 dilution step	Limited effect
G	0/23	0	No detectable effect
E	21/23	1 dilution step	Uniform but small effect
GE	7/23	1 dilution step	Limited effect
MG	3/23	1 dilution step	Limited effect
ME	2/23	1 dilution step	Limited effect
MGE	0/23	0	No detectable effect

No treatment achieved a ≥4-fold MIC reduction, which was the prespecified operational threshold for a substantial CCCP-responsive effect.

**Table 4:** Assembly metrics for eight representative sequenced derivatives selected to illustrate temporal stability under marbofloxacin and MGE exposure.

Sample	Passage Condition	Contigs	Total Length (bp)	N_50_	GC (%)
E29	Day 0 control replicate 1	531	6,781,215	23,927	66.25
E30	Day 0 control replicate 2	528	6,782,775	25,805	66.25
E37	Day 2 M	434	6,782,414	32,761	66.25
E31	Day 2 MGE	469	6,783,315	29,417	66.25
E28	Day 6 M	519	6,780,590	25,599	66.25
E22	Day 6 MGE	460	6,781,635	30,565	66.24
E21	Day 10 M	551	6,781,837	23,868	66.25
E15	Day 10 MGE	479	6,782,450	28,917	66.25

**Table 5:** Consolidated genome-screening findings relevant to interpretation of the observed phenotypic trajectories.

Feature Category	Main Observation	Treatment Linkage	Interpretive Value
The number of detected resistance genes	46–47 genes/sample	Minimal	No broad resistome expansion
Fluoroquinolone-associated loci	Mostly stable coverage/identity	Minimal	No pervasive treatment-specific remodeling
Point mutations	Candidate *oprN* c.311A>G variant resulting in p.Asn104Ser	Isolated event	Insufficient to explain overall phenotype alone
Plasmid/mobile genetic element/prophage context	Detected, but without a clear treatment-stratified pattern explaining minimum inhibitory concentration trajectories	Weak	Supports cautious interpretation
Virulome	Largely conserved with scattered sample-specific absences	Weak	No coherent phenotype-defining signature

## Data Availability

The whole-genome sequencing data generated in this study have been submitted to the National Center for Biotechnology Information (NCBI) under BioProject accession PRJNA1499240. The 23 corresponding BioSample records are available under accession numbers SAMN61871536–SAMN61871558. The draft genome assemblies have been submitted under genome submission SUB16350449. Phenotypic datasets supporting the findings of this study are included in the article and its Supplementary Materials.

## Supplementary Materials

The following supporting information can be downloaded at: https://www.mdpi.com/article/10.3390/antibiotics15080754/s1. Table S1: Complete 10-day minimum inhibitory concentration (MIC) and fractional inhibitory concentration (FIC) trajectory dataset for serial passaging. Table S2: Isolate-level MICs determined in the absence and presence of CCCP in 22 canine clinical isolates and the *Pseudomonas aeruginosa* ATCC 27853 reference strain. Table S3: Comparison of planktonic MICs and MTS-derived relative EC_50_ values for *Pseudomonas aeruginosa* ATCC 27853. Table S4: Sequencing and assembly metrics for the genomes included in the final analysis. Table S5: Summary of fluoroquinolone-associated resistance determinants, candidate variants, plasmid-associated contigs, mobile genetic elements, prophages, and virulence-associated genes identified among the sequenced derivatives.
