# Supplementary material for: Combined Marbofloxacin-Geraniol-Tris-EDTA Exposure Delays Marbofloxacin Resistance Escalation in Pseudomonas aeruginosa While Early Genomic Changes Remain Limited
Source: Antibiotics (Basel). 2026 Aug 5;15(8):754. doi: 10.3390/antibiotics15080754 (PMC13510036; doi:10.3390/antibiotics15080754)
Supplement: Supplementary file 1 [file antibiotics-15-00754-s001.zip › antibiotics-4486043-supplementary.pdf]

Supplementary Table S1. Complete 10-day minimum inhibitory concentration (MIC) and fractional inhibitory concentration (FIC) trajectory dataset for serial passaging. Daily MIC values are shown for marbofloxacin (M), geraniol (G), Tris-EDTA (E), and the double and triple combinations. FIC index categories were assigned as follows: <0.5, synergy; 0.5-1.0, additivity/partial synergy; >1.0-4.0, indifference; >4.0, antagonism. Combination MICs are expressed as component concentrations in the order indicated in the column header.

Day	M MIC	G MIC	E MIC	ME MIC	ME FIC	ME class	GE MIC	GE FIC	GE class	MG MIC	MG FIC	MG class	MGE MIC	MGE FIC	MGE class
1	2	1.6	0.8	0.25+0.5	0.75	Additivity/partial synergy	0.4+0.4	0.75	Additivity/partial synergy	1+1	1.125	Indifference	0.25+0.287+0.325	0.711	Additivity/partial synergy
2	2	1.6	1.6	0.25+0.5	0.438	Synergy	0.4+0.4	0.5	Additivity/partial synergy	1+1	1.125	Indifference	0.125+0.1237+0.1625	0.241	Synergy
3	4	1.6	1.6	0.5+1	0.75	Additivity/partial synergy	0.8+0.8	1.0	Additivity/partial synergy	2+2	1.75	Indifference	0.125+0.1237+0.1625	0.21	Synergy
4	4	1.6	1.6	0.5+1	0.75	Additivity/partial synergy	0.2+0.2	0.25	Synergy	1+1	0.875	Additivity/partial synergy	0.25+0.287+0.325	0.891	Additivity/partial synergy
5	4	3.2	1.6	0.5+1	0.75	Additivity/partial synergy	0.8+0.8	0.75	Additivity/partial synergy	2+2	1.125	Indifference	0.125+0.1237+0.1625	0.171	Synergy
6	4	1.6	1.6	0.25+0.5	0.375	Synergy	0.8+0.8	1.0	Additivity/partial synergy	2+2	1.75	Indifference	0.125+0.1237+0.1625	0.21	Synergy
7	8	3.2	1.6	0.25+0.5	0.344	Synergy	0.8+0.8	0.75	Additivity/partial synergy	2+2	0.875	Additivity/partial synergy	0.25+0.287+0.325	0.324	Synergy
8	16	1.6	1.6	0.25+0.5	0.328	Synergy	0.8+0.8	1.0	Additivity/partial synergy	4+4	2.75	Indifference	0.125+0.1237+0.1625	0.187	Synergy
9	16	1.6	1.6	0.25+0.5	0.328	Synergy	0.8+0.8	1.0	Additivity/partial synergy	4+4	2.75	Indifference	0.25+0.287+0.325	0.398	Synergy
10	16	1.6	1.6	0.5+1	0.656	Additivity/partial synergy	0.8+0.8	1.0	Additivity/partial synergy	2+2	1.375	Indifference	0.25+0.287+0.325	0.797	Additivity/partial synergy

Table S2. Isolate-level MICs determined in the absence and presence of CCCP in 22 canine clinical isolates and the *Pseudomonas aeruginosa* ATCC 27853 reference strain. Values are presented for each single agent and combination regimen.

ISOLATE	M -CCCP	M +CCCP	G -CCCP	G +CCCP	E -CCCP	E +CCCP	GE -CCCP	GE +CCCP	MG -CCCP	MG +CCCP	ME -CCCP	ME +CCCP	MGE -CCCP	MGE +CCCP
1	2	2	1.6	1.6	1.6	0.8	0.8+0.8	0.4+0.4	1+1	1+1	0.25+0.5	0.25+0.5	0.25+0.2875+0.325	0.25+0.2875+0.325
2	4	4	1.6	1.6	1.6	0.8	0.8+0.8	0.8+0.8	2+2	2+2	0.5+1	0.5+1	0.125+ 0.1237+ 0.1625	0.25+0.2875+0.325
3	2	2	1.6	1.6	1.6	0.8	0.8+0.8	0.4+0.4	1+1	1+1	0.25+0.5	0.25+0.5	0.125+ 0.1237+ 0.1625	0.25+0.2875+0.325
4	2	2	1.6	1.6	1.6	0.8	0.8+0.8	0.4+0.4	1+1	1+1	0.25+0.5	0.25+0.5	0.25+0.2875+0.325	0.25+0.2875+0.325
5	4	2	1.6	1.6	1.6	0.8	0.8+0.8	0.4+0.4	1+1	1+1	0.25+0.5	0.25+0.5	0.25+0.2875+0.325	0.25+0.2875+0.325
6	2	2	1.6	1.6	1.6	0.8	0.8+0.8	0.4+0.4	1+1	1+1	0.25+0.5	0.25+0.5	0.25+0.2875+0.325	0.25+0.2875+0.325
7	2	2	1.6	1.6	1.6	0.8	0.8+0.8	0.4+0.4	1+1	1+1	0.25+0.5	0.25+0.5	0.25+0.2875+0.325	0.25+0.2875+0.325
8	2	2	1.6	1.6	1.6	0.8	0.8+0.8	0.4+0.4	1+1	1+1	0.25+0.5	0.25+0.5	0.25+0.2875+0.325	0.25+0.2875+0.325
9	4	4	1.6	1.6	1.6	0.8	0.8+0.8	0.8+0.8	2+2	2+2	0.5+1	0.5+1	0.25+0.2875+0.325	0.25+0.2875+0.325
10	1	1	1.6	1.6	1.6	0.8	0.8+0.8	0.8+0.8	1+1	1+1	0.5+1	0.5+1	0.25+0.2875+0.325	0.25+0.2875+0.325
11	2	4	1.6	1.6	1.6	0.8	0.8+0.8	0.8+0.8	1+1	1+1	0.25+0.5	0.25+0.5	0.25+0.2875+0.325	0.25+0.2875+0.325
12	2	2	1.6	1.6	1.6	1.6	0.8+0.8	0.8+0.8	2+2	2+2	0.25+0.5	0.25+0.5	0.25+0.2875+0.325	0.25+0.2875+0.325
13	4	4	1.6	1.6	1.6	0.8	0.8+0.8	0.8+0.8	2+2	2+2	0.5+1	0.5+1	0.25+0.2875+0.325	0.25+0.2875+0.325
14	1	1	1.6	1.6	1.6	0.8	0.8+0.8	0.8+0.8	1+1	0.5+0.5	0.25+0.5	0.25+0.5	0.125+ 0.1237+ 0.1625	0.125+ 0.1237+ 0.1625
15	2	1	1.6	1.6	1.6	0.8	0.8+0.8	0.8+0.8	1+1	1+1	0.25+0.5	0.25+0.5	0.25+0.2875+0.325	0.25+0.2875+0.325
16	4	2	1.6	1.6	1.6	0.8	0.8+0.8	0.8+0.8	2+2	1+1	0.25+0.5	0.25+0.5	0.125+ 0.1237+ 0.1625	0.25+0.2875+0.325
17	1	1	1.6	1.6	1.6	0.8	0.8+0.8	0.8+0.8	1+1	1+1	0.25+0.5	0.25+0.5	0.25+0.2875+0.325	0.25+0.2875+0.325
18	2	2	1.6	1.6	1.6	0.8	0.8+0.8	0.8+0.8	2+2	1+1	0.25+0.5	0.5+1	0.25+0.2875+0.325	0.25+0.2875+0.325
19	4	4	1.6	1.6	1.6	0.8	0.8+0.8	0.8+0.8	2+2	2+2	0.25+0.5	0.25+0.5	0.25+0.2875+0.325	0.25+0.2875+0.325
20	8	4	1.6	1.6	1.6	0.8	0.8+0.8	0.8+0.8	2+2	2+2	0.25+0.5	0.25+0.5	0.25+0.2875+0.325	0.25+0.2875+0.325
21	2	1	1.6	1.6	1.6	1.6	0.8+0.8	0.8+0.8	1+1	1+1	0.25+0.5	0.25+0.5	0.25+0.2875+0.325	0.25+0.2875+0.325
22	8	8	1.6	1.6	1.6	0.8	0.8+0.8	0.8+0.8	4+4	4+4	0.5+1	0.25+0.5	0.25+0.2875+0.325	0.25+0.2875+0.325
23	16	16	1.6	1.6	1.6	0.8	0.8+0.8	0.8+0.8	4+4	4+4	0.5+1	0.25+0.5	0.25+0.2875+0.325	0.25+0.2875+0.325

Table S3. Comparison of planktonic MICs and MTS-derived relative EC_{50} values for *Pseudomonas aeruginosa* ATCC 27853. For combination regimens, concentrations are presented for the individual components in the order specified in the treatment abbreviation. The rEC_{50}/MIC ratio refers to the corresponding dilution level.

Treatment	Planktonic MIC	MTS-based EC_{50}	EC_{50}/MIC
M	2	2	1.00
G	1.6	1.6	1.00
E	0.8	0.8	1.00
GE	0.4+0.4	0.4+0.4	1.00
MG	1+1	1+1	1.00
ME	0.25+0.5	0.25+0.5	1.00
MGE	0.25+0.287+0.325	0.25+0.287+0.325	1.00

Table S4. Sequencing and assembly metrics for the genomes included in the final analysis. This table summarizes the complete set of sequenced *Pseudomonas aeruginosa* ATCC 27853 derivatives.

Sample	Sampling point	Treatment	Reads	# contigs	Total length (bp)	N50 (bp)	GC (%)	Resistance genes (CARD)
E29	0 day	Control	1,724,056	531	6,781,215	23,927	66.25	47
E30	0 day	Control	1,893,978	528	6,782,775	25,805	66.25	46
E19	10 days	E	2,523,613	428	6,780,895	34,918	66.24	47
E20	10 days	G	2,412,332	515	6,786,783	30,477	66.23	47
E16	10 days	GE	1,635,010	566	6,780,674	23,538	66.26	47
E21	10 days	M	1,697,201	551	6,781,837	23,868	66.26	47
E17	10 days	ME	1,505,370	576	6,782,600	25,117	66.25	47
E18	10 days	MG	1,862,597	519	6,786,365	25,823	66.25	46
E15	10 days	MGE	2,104,031	479	6,782,450	28,917	66.25	47
E35	2 days	E	3,339,657	398	6,781,769	38,174	66.24	47
E36	2 days	G	1,644,197	562	6,788,158	23,156	66.26	47
E34	2 days	GE	2,343,282	425	6,781,279	35,751	66.25	47
E37	2 days	M	2,702,448	434	6,782,414	32,761	66.25	47
E32	2 days	ME	2,110,435	456	6,782,265	33,232	66.25	47
E33	2 days	MG	2,354,248	465	6,775,507	29,683	66.24	46
E31	2 days	MGE	2,029,568	469	6,783,315	29,417	66.25	47
E26	6 days	E	1,916,050	499	6,781,158	27,704	66.25	47
E27	6 days	G	2,944,147	473	6,799,313	34,658	66.16	47
E25	6 days	GE	2,243,403	461	6,780,542	35,246	66.25	47
E28	6 days	M	1,707,184	519	6,780,590	25,599	66.25	47
E24	6 days	ME	1,787,106	533	6,783,377	28,910	66.25	47
E23	6 days	MG	1,849,865	514	6,780,466	26,899	66.24	47
E22	6 days	MGE	2,432,547	460	6,781,635	30,565	66.24	46

Table S5. Summary of fluoroquinolone-associated resistance determinants, candidate variants, plasmid-associated contigs, mobile genetic elements, prophages, and virulence-associated genes identified among the sequenced derivatives.

Feature class	Finding	Sample(s)	Interpretive note
Fluoroquinolone-associated resistome screen	19 determinants screened across sequenced derivatives (ArmR, MexA/B/C/D/E/F/V/W/Y, OprJ/N, ParR/S, PmpM, CpxR, SoxR, RsmA, YajC)	All sequenced samples	Broad conservation of the known fluoroquinolone-associated resistome
Determinants excluded from mutation follow-up	*armR*, *cpxR*, *soxR*, and *mexE* showed 100% coverage and 100% identity to the CARD reference	All sequenced samples	No treatment-linked sequence divergence inferred for these loci
Partially covered determinant	*mexY* showed incomplete coverage and was therefore not interpreted for point-mutation analysis	All sequenced samples	Conservative exclusion from mutation-level inference
Nonsynonymous variant detected	*oprN* A→C substitution with predicted Ser→Arg change	E32 (day 2, ME)	Only treatment-linked nonsynonymous change highlighted in the manuscript
Plasmid-associated fluoroquinolone determinant	*armR* detected on a predicted plasmid background	E22 (day 6, MGE)	Supports occasional mobile-element association without broad restructuring of the resistome
Insertion sequence-associated determinant	*yajC* repeatedly associated with insertion-sequence content (Psy29)	Multiple sequenced samples	Suggests mobile-element proximity for one fluoroquinolone-associated locus
Other mobile-element-associated loci	*muxA*, *muxB*, *muxC*, and *opmB* associated with Pa2 insertion-sequence content	Multiple sequenced samples	Mobile elements were present but did not coincide with extensive treatment-linked genome divergence
Prophage content	65 intact prophages identified in total	Across sequenced samples	Prophage carriage was common but not obviously treatment-specific in the main interpretation
Virulence-factor profile	Core virulence repertoire largely preserved with only limited sample-specific absences noted in exploratory screening	Across sequenced samples	Supports overall genomic stability during short-term passaging
